# Cardiopulmonary resuscitation training, knowledge and basic life support practice among church laypersons in Johannesburg, South Africa

**DOI:** 10.4102/safp.v68i1.6359

**Published:** 2026-10-06

**Authors:** Michael Ibanbeteliehe Ihwo, Martin Botha, Veronica Akwenabuaye Undelikwo, Sunday Oladapo Sofola-Orukotan

**Affiliations:** 1 Division of Emergency Medicine, Faculty of Health Sciences, University of the Witwatersrand, Johannesburg, South Africa; 2 Department of Sociology, Faculty of Social Sciences, University of Calabar, Calabar, Nigeria; 3 Departments of Emergency Medicine, Mackay Base Hospital, Mackay, Queensland, Australia

**Keywords:** basic life support, cardiac arrest, cardiopulmonary resuscitation, church attendees, CPR knowledge, out-of-hospital cardiac arrest, resuscitation training, South Africa

## Abstract

**Background:**

Out-of-hospital cardiac arrest (OHCA) is the most common cause of death globally, and therefore it is a serious public health issue. The chance of survival is increased when bystander cardiopulmonary resuscitation (CPR) is incorporated into the OHCA chain of survival. The aim is to determine if there is an association between exposure to CPR training and basic life support (BLS) practice among laypersons in a church community.

**Methods:**

At a church community in Braamfontein, South Africa, a quantitative analytical cross-sectional study of the congregation was carried out using convenience sampling. Data were collected using a structured questionnaire, and descriptive and bivariate analyses were conducted.

**Results:**

Only 14.6% of respondents had received CPR training. Cardiopulmonary resuscitation training was significantly associated with the ability to effectively open the airway (*p* = 0.001), perform mouth-to-mouth respiration (*p* = 0.001), perform chest compression (*p* = 0.013), perform both mouth-to-mouth ventilation and chest compression (*p* = 0.001), perform CPR effectively (*p* = 0.003) and willingness to learn CPR (*p* = 0.029).

**Conclusion:**

Although the church laypersons’ knowledge of CPR was poor, participants with prior CPR training reported being better at performing a variety of CPR techniques. With about one-fourth of respondents reporting having witnessed a cardiac arrest, it is important to proactively investigate and put into practice various forms of layperson CPR training as a strategy to ensure the laypeople are equipped to help increase the survival rates of OHCA.

**Contribution:**

The study enhances our understanding of how CPR training among laypersons in the church can increase their willingness to administer BLS and identifies churches as key locations for community CPR capacity building.

## Introduction

Sudden unexpected cardiac arrest (CA) is a medical emergency that needs to be recognised and managed quickly to increase the chances of survival.^1^ Out-of-hospital cardiac arrest (OHCA) is one of the leading issues of global public health significance. An annual OHCA incidence of 110.8 per 100 000 population has been reported in Europe, and between 67 and 170 per 100 000 in the United States (US).^2,3^ The survival rates of OHCA vary between regions but average at approximately 10%.^2,4^ Yuyun et al. found that cardiovascular diseases (CVDs) were responsible for 37% of all non-communicable illnesses and 13% of all fatalities in sub-Saharan Africa (SSA), despite the lack of data on the epidemiology of OHCA in SSA.^5^ Additionally, they predicted that in a few decades, communicable diseases will be surpassed by CVDs and other non-communicable diseases (NCDs) as SSA’s primary cause of death. A study carried out in Douala, Cameroon, revealed an incidence rate of 24.2 sudden cardiac deaths per 100 000 people, with OHCA accounting for 58.6% of these instances.^6^ Risk factors for sudden cardiac arrest (SCA), including diabetes, hypertension, ischaemic heart disease and other heart conditions, were among the top 10 underlying natural causes of mortality in South Africa in 2016, according to Statistics South Africa.^7^ South Africa has a significant prevalence of SCA risk factors and CVD, according to other studies.^8,9^

After an OHCA, bystanders significantly improve both survival and neurological outcomes. The metaphorical ‘chain of survival’ connects a number of basic life support (BLS) procedures that, when carried out quickly after a cardiac arrest (CA), may have the most impact on mortality reduction. The first link in the chain of survival is ‘prearrival care’, which is defined as basic first responder interventions carried out by onlookers prior to the arrival of qualified emergency medical personnel.^10^ These include the use of an automated external defibrillator (AED), prompt bystander cardiopulmonary resuscitation (CPR) and early detection of CA symptoms and emergency response system (ERS) activation. Out-of-hospital cardiac arrest survival has increased by more than two times when onlookers carry out these actions appropriately.^11,12^ Since CPR without an AED shock is never effective in shockable CA dysrhythmias, the added use of an AED can further increase survivability.^13^ Nevertheless, early performance of bystander CPR is necessary in such cases to allow enough time for myocardial perfusion because the AED may not be available in most public venues in low-to-middle income nations. For every minute that a patient with SCA has experienced ventricular fibrillation (VF) without receiving CPR, their chances of survival drop by 7% to 10%.^14^ The window of opportunity for successful defibrillation is extended when CPR is administered right away because compressions maintain the amplitude of the VF.

The rate of bystander CPR application is still low, despite the overwhelming data supporting its critical necessity.^15^ Bystander CPR rates vary widely throughout the world, with estimates ranging from 1% to 74%.^16,17^ The disparities in bystander CPR execution may be caused, among other things, by a lack of awareness about its benefits and the unavailability of the AED. Even though laypeople observed 86.2% of OHCA, only 7.4% of them received bystander CPR, according to Cameroon research.^6^ Even though 68% of OHCA cases were witnessed, bystander CPR was only administered in 36% of cases, according to a retrospective analysis of OHCA patients seen by emergency medical service (EMS) workers in the Johannesburg metropolitan region over a 7-year period.^18^

Life-threatening emergencies can occur anywhere, at any moment. Lack of confidence and expertise could lead to inaction, which could have disastrous effects and possibly even legal ramifications.^19^ Since anyone can have an SCA, everyone should be trained in basic CPR.^1^ The percentage of laypeople who have received CPR training varies by nation.^17,20,21,22,23,24^ Community-based training has enhanced OHCA survival by raising awareness, usefulness and implementation rates.^4,10,23,25^ There is a significant chance of saving lives if a greater percentage of the community has adequate CPR knowledge, skills and training because OHCA is primarily witnessed by bystanders. Numerous international studies have been carried out to determine laypeople’s overall understanding and attitudes on BLS after CA.^10,19,26,27^ Ojifinni et al. found that student-teachers at a South African university had low knowledge about CPR and a positive attitude (willingness to learn how to perform CPR), despite the paucity of data from that country.^24^

Some researchers have investigated how the general public views and practices BLS. According to a Jordanian study, most participants were eager to do CPR on family members, but their main worry was that they might make a mistake.^10^ Fear of hurting the victim, anxiety about mouth-to-mouth breathing, and the possibility of catching an infection are additional reasons why people were reluctant to do CPR. Other limiting factors included inadequate information or training, fear of litigation, lack of confidence, lack of skill and fear of the victim’s reaction after regaining consciousness after mouth-to-mouth breathing.^9,14^

A variety of tactics, including mass training, media use and video self-instruction, have been used as educational methods to reach a large number of laypeople and enhance their psychomotor abilities and understanding of BLS.^8^ Television shows and films are common sources of information about BLS.^10^ Regular evaluation of laypeople’s knowledge and attitudes about BLS may assist in uncovering obstacles to performing CPR, given the high prevalence of risk factors for CVDs and SCA, the high rate of observed OHCA, and the low rates of bystander CPR in South Africa. This study aimed to assess the association between exposure to CPR training and the practice of BLS among laypeople in a Johannesburg church community.

## Research methods and design

### Study design

This study adopted a quantitative analytical cross-sectional survey design and was carried out over a 4-month period, from 06 December 2020 to 28 March 2021. Data were collected from each respondent at one point in time. The use of this design is appropriate for this study as it allowed the researchers to test hypotheses and establish relationships between variables.

### Setting

The study was carried out at a church community in Braamfontein, Johannesburg, South Africa. The site was chosen as it consists of worshippers from various locations in Johannesburg as well as people from diverse backgrounds, including the universities in Johannesburg. This diversity made the church community an ideal place to assess the association between exposure to CPR training and the practice of BLS among laypersons.

### Sample size

The minimum sample size was determined using the Kish Leslie (1965) formula, with a standard normal deviate of 1.96 at the 95% confidence level, an estimated adequate knowledge of 20%,^28^ *p* = 0.20, *q* = 0.80 and a margin of error of 0.05. The calculated sample size was 246. After adjusting for a 10% non-response rate, the sample size was increased to 270. However, 300 respondents were approached.

### Study population and sampling strategy

Eligible respondents were adult worshippers aged 18 years old and older who were present and approachable immediately after Sunday Mass services at 07:30, 09:30 and 18:00. Exclusion criteria included medical or paramedical personnel and health sciences students to focus on lay members without professional BLS training. Convenience sampling was used as it prioritised feasibility in a church setting where random sampling frames (e.g. parish registers) were unavailable and rapid post-mass dispersal limited structured approaches. Researchers stationed at the church exit approached every eligible worshipper who was willing to participate during the data collection period until the target was met. Of the 300 worshippers approached, 220 (73.3%) consented and completed questionnaires (response rate: 73.3%), with non-respondents primarily citing time constraints and lack of interest. Although convenience sampling limits generalisation beyond the church population, the high response rate, consistent recruitment across multiple mass services and a focused setting enhance internal validity for context-specific insights.

### Data collection

Data were collected through self-administered, closed-ended paper questionnaires distributed immediately after Mass in a quiet area adjacent to the church exit. The pastor informed the church members about the study, and then eligible worshippers were approached at the church premises and were given an informed consent form and a detailed explanation about the nature of the study. Researchers provided brief instructions, answered questions and retrieved completed questionnaires on-site to minimise loss. The questionnaire assessed demographic information, respondents’ knowledge, attitude and practice of BLS. Some questions were adapted from two previously validated questionnaires,^29,30^ while others were incorporated from a questionnaire based on the latest American Heart Association (AHA) guidelines on BLS.^21^ After pre-test among 20 adult worshippers in another church in Johannesburg, the final questionnaire was modified based on the pre-test feedback and content validity determined based on expert review by two emergency medicine specialists, a formal reliability test was not conducted.

### Measurement of variables

Knowledge of CPR was assessed using two items that measured respondents’ understanding of the meaning of CPR and when CPR should be administered. Training on CPR was measured using a dichotomous item by asking respondents whether they had received CPR training. Responses were coded as ‘Yes’ or ‘No’ and were used in the analysis to classify respondents as CPR trained and CPR untrained, respectively.

### Data analysis

Completed questionnaires were manually entered into Microsoft Excel and imported into Stata Version 18 (Stata Corp LLC, College Station, Texas, United States). Descriptive and bivariate analyses were conducted. The sociodemographic variables were described using percentages for categorical variables and mean ± standard deviation (s.d.) for continuous variables. Bar graphs and pie charts were used to illustrate categorical variables. The participants were characterised and compared according to the detection of CA, their comprehension of practical CPR techniques and their BLS skills, stratified by prior CPR training using Pearson’s chi-squared and Fisher’s exact tests when applicable. A two-sided *p*-value of less than 0.05 was considered statistically significant.

### Ethical considerations

An application for full ethical approval was made to theHuman Research Ethics Committee (Medical), University of the Witwatersrand and ethics consent was received on 05 June 2020. The ethics approval number is M200538 MED-20-04-118. Prior to the distribution of the questionnaire, written informed consent was obtained. Those involved were assured of anonymity, voluntary withdrawal at any time and data confidentiality. No incentives were offered.

## Results

The data from 205 participants were analysed, excluding 15 incomplete questionnaires. The participants’ average age was 36.6 ± 12.6, with most being below 40 years old − 128 (62.4%). Half of the participants were female (*n* = 104; 50.7%), making the cohort equally divided between the sexes. Of the participants, 101 (49.3%) were single, and 181 (88.3%) of the participants had completed education higher than primary school (Table 1).

**TABLE 1:** Study participants’ demographic data (*N* = 205).

Variables	*n*	%
Age in years	-	-
**Age group in years**	-	-
< 40	128	62.4
≥ 40	77	37.6
**Sex**	-	-
Female	104	50.7
Male	101	49.3
**Marital status**	-	-
Single	101	49.3
Married	78	38.1
Divorced	21	10.2
Widowed	5	2.4
**Educational level**	-	-
Primary school	24	11.7
Matriculation	65	31.7
Diploma	43	21.0
University	40	19.5
Postgraduate	33	16.1
**Employment sector**	-	-
Government	18	8.8
Private	52	25.4
Self-employed	59	28.8
Student	36	17.6
Unemployed	31	15.0
Retired	9	4.4

Note: The mean ± s.d. for age is 36.6 ± 12.6.

### Knowledge of CPR and AED among participants with and without previous CPR training

Table 2 highlights the participants’ knowledge of CPR and AED based on previous CPR training. Among respondents who had received CPR training, approximately eight in 10 (80.9%) did not know the meaning of the term CPR. Among respondents who had received CPR training, knowledge of when to administer CPR was as follows: Loss of consciousness (61.5%), gasping and not breathing (55.1%), not moving (17.6%), no response to a call (63.9%) and no response to touch (66.3%). Knowledge of AEDs was low, with only 26.8% of participants having heard of an AED. Similarly, only 11.7% reported having heard of it but not having seen it, and thought it was a device to support breathing, while only 11.2% correctly identified it as a device used to restore a normal heart rhythm during CA. Cardiopulmonary resuscitation-trained respondents were slightly more knowledgeable about the assessment of an AED. Previous training on CPR was significantly associated with knowing when to give someone CPR when there is no movement (*p* = 0.014) and no response to a call (*p* = 0.005). No significant associations were observed between previous CPR training and other CPR and AED knowledge variables.

**TABLE 2:** Effect of prior cardiopulmonary resuscitation training on knowledge variables.

Knowledge variable	Total^†^		CPR training				*p*
			CPR-trained^‡^		CPR-untrained^§^		
	*n*	%	*n*	%	*n*	%	
**CPR meaning**	-	-	-	-	-	-	< 0.001*
Incorrect	165	80.9	12	40.0	153	87.9	-
Correct	39	19.1	18	60.0	21	12.1	-
**How would you know when to give someone CPR – Loss of consciousness**	-	-	-	-	-	-	0.820
No	79	38.5	11	36.7	68	38.9	-
Yes	126	61.5	19	63.3	107	61.1	-
**How would you know when to give someone CPR – Gasping and not breathing**	-	-	-	-	-	-	0.328
No	92	44.9	11	36.7	81	46.3	-
Yes	113	55.1	19	63.3	94	53.7	-
**How would you know when to give someone CPR – Individual not moving**	-	-	-	-	-	-	0.014*
No	169	82.4	20	66.7	149	85.1	-
Yes	36	17.6	10	33.3	26	14.9	-
**How can loss of consciousness be determined – No response when called**	-	-	-	-	-	-	0.005*
No	74	36.1	4	13.3	70	40.0	-
Yes	131	63.9	26	86.7	105	60.0	-
**How can loss of consciousness be determined – No response when touched**	-	-	-	-	-	-	0.087
No	69	33.7	6	20.0	63	36.0	-
Yes	136	66.3	24	80.0	112	64.0	-
**How can loss of consciousness be determined – Not moving at all**	-	-	-	-	-	-	0.304
No	120	58.5	15	50.0	105	60.0	-
Yes	85	41.5	15	50.0	70	40.0	-
**What is an AED? Never heard of it**	-	-	-	-	-	-	< 0.001*
Heard of it	55	26.8	21	70.0	34	19.4	-
Never heard of it	150	73.2	9	30.0	141	80.6	-
**What is an AED? Heard but not seen it**	-	-	-	-	-	-	0.764
No	181	88.3	26	86.7	155	88.6	-
Yes	24	11.7	4	13.3	20	11.4	-
**What is an AED? Device to support breathing**	-	-	-	-	-	-	0.764
No	181	88.3	26	86.7	155	88.6	-
Yes	24	11.7	4	13.3	20	11.4	-
**What is an AED? A device to restart the heart**	-	-	-	-	-	-	< 0.001*
No	182	88.8	17	56.7	165	94.3	-
Yes	23	11.2	13	43.3	10	5.7	-

CPR, cardiopulmonary resuscitation; AED, automated external defibrillator.

† , *N* = 205 (100%);

‡ , *N* = 30 (14.6%);

§ , *N* = 175 (85.4%).

* , Significant at *p* < 0.05.

### CPR ability

Table 3 displays the participants’ responses about their perceived capacity to administer CPR. Most participants said they were capable of performing chest compressions (80% of those trained in CPR versus 56% of those untrained). Also, 56.7% of the CPR-trained participants and 10.9% of the CPR-untrained participants reported that they could effectively open the airways. Furthermore, 76.7% of CPR-trained respondents and 25.1% of CPR-untrained respondents indicated that they could perform mouth-to-mouth ventilation. Eighty per cent of CPR-trained participants and 26.3% of CPR-untrained participants stated that they could perform both mouth-to-mouth ventilation and chest compression. Additionally, 50% of CPR-trained respondents were confident in their ability to perform CPR effectively. Many of the participants said they would be willing to learn CPR. Ability to effectively open the airway (*p* = 0.001), perform ventilation from mouth to mouth (*p* = 0.001), perform chest compression (*p* = 0.013), do both chest compression and ventilation from mouth to mouth (*p* = 0.001), perform CPR effectively (*p* = 0.003), and willingness to learn CPR (*p* = 0.029) were significantly associated with CPR training.

**TABLE 3:** Respondents’ perceived ability to perform cardiopulmonary resuscitation stratified by cardiopulmonary resuscitation-trained and cardiopulmonary resuscitation-untrained.

CPR ability	Total^†^		CPR training				*p*
			CPR-trained^‡^		CPR-untrained^§^		
	*n*	%	*n*	%	*n*	%	
**Effectively open the airway**	-	-	-	-	-	-	**< 0.001**
No	169	82.4	13	43.3	156	89.1	-
Yes	36	17.6	17	56.7	19	10.9	-
**Perform mouth to mouth ventilation**	-	-	-	-	-	-	**< 0.001**
No	138	67.3	7	23.3	131	74.9	-
Yes	67	32.7	23	76.7	44	25.1	-
**Perform chest compression**	-	-	-	-	-	-	**0.013**
No	83	40.5	6	20	77	44	-
Yes	122	59.5	24	80	98	56	-
**Perform both mouth to mouth ventilation and chest compression**	-	-	-	-	-	-	**< 0.001**
No	135	65.9	6	20	129	73.7	-
Yes	70	34.1	24	80	46	26.3	-
**Able to perform CPR effectively**	-	-	-	-	-	-	**0.003**
No	149	72.7	15	50	134	76.6	-
Yes	56	27.3	15	50	41	23.4	-
**Willingness to learn CPR**	-	-	-	-	-	-	0.029*
No	21	10.2	7	23.3	14	8.0	-
Not sure	31	15.1	2	6.7	29	16.6	-
Yes	153	74.7	21	70.0	132	75.4	-

Note: Bold values, significant at *p* < 0.05.

CPR, cardiopulmonary resuscitation.

† , *N* = 205 (100%);

‡ , *N* = 30 (14.6%);

§ , *N* = 175 (85.4%).

* , CPR, Fisher’s Exact test.

### CPR training status

The percentage of participants who had received CPR training is displayed in Figure 1. Of the participants, about 30 (14.6%) had received prior CPR training.

**FIGURE 1:**
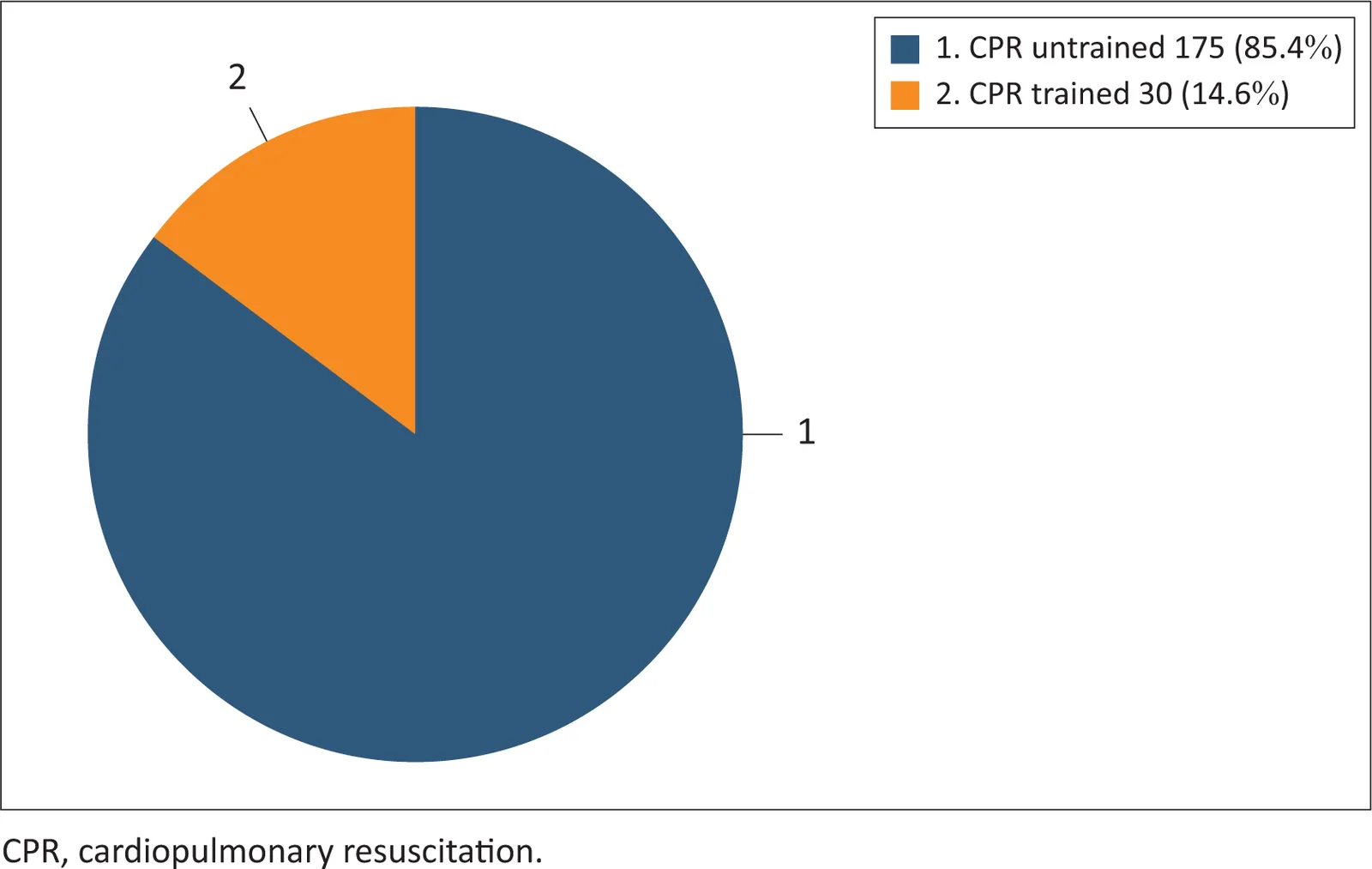
Pie chart displaying the participants’ proportion by cardiopulmonary resuscitation training status (*N* = 205).

### CPR training and having witnessed a cardiac arrest

Figure 2 displays the proportion of respondents who had been present at a CA. Thirteen (43.3%) of those trained and 80 (45.7%) of the CPR-untrained participants reported having witnessed a CA.

**FIGURE 2:**
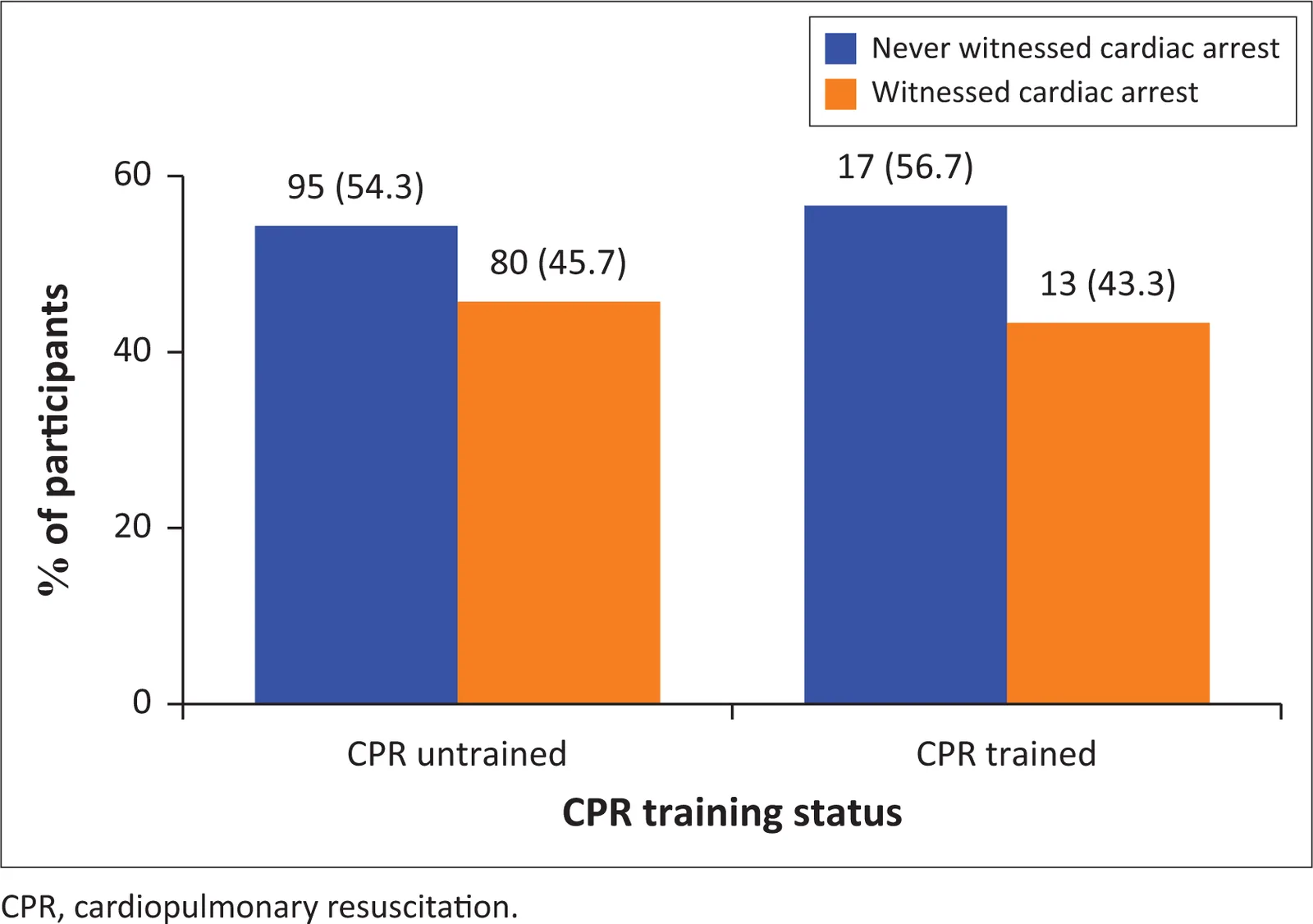
Respondents who witnessed cardiac arrest, by cardiopulmonary resuscitation training status.

### Participants’ concerns regarding CPR

Fear of committing an error, contracting a communicable disease, and harming organs were the top three fears when conducting CPR on strangers and family members or relatives, in that order (Figure 3).

**FIGURE 3:**
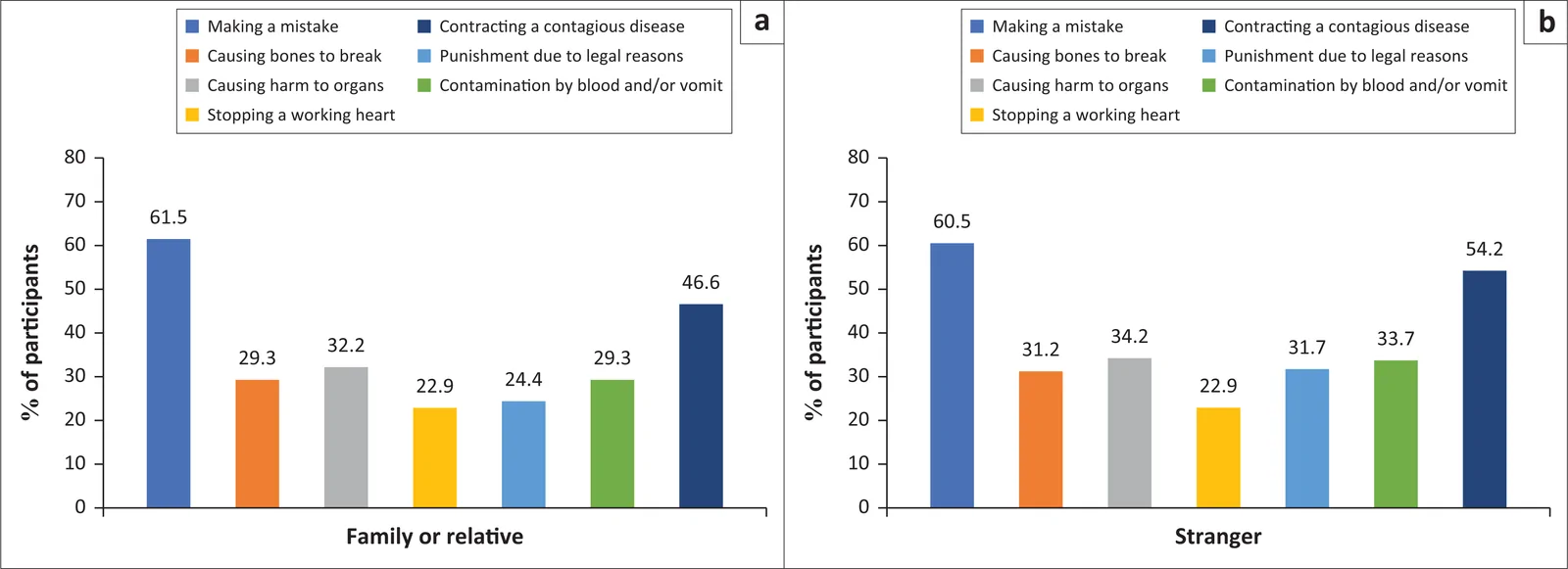
Reasons for unwillingness to perform cardiopulmonary resuscitation: (a) family or relative and (b) stranger.

## Discussion

There is a significant chance of saving lives if a greater percentage of the community has adequate CPR knowledge, skills, and training because OHCA is primarily witnessed by bystanders. The study assessed the effect of participants’ previous CPR training on knowledge of CPR and AED, perceived ability to perform CPR, attitudes towards CPR and the reasons behind not performing it.

Less than a quarter (14.6%) of individuals had received prior CPR training, according to the study; Ojifinni et al. reported a lower proportion (8.9%) in a study conducted among student-teachers in a tertiary institution in South Africa.^24^ However, higher rates have been reported in some international studies: China 34.8%,^17^ Ukraine 53%,^20^ Jordan 29%,^21^ New Zealand 74%^22^ and Slovenia 69.4%.^27^ In order to keep up with global trends, public CPR training needs to be increased in South Africa. Less than one-third of the total respondents reported being able to effectively provide CPR. The significance of current, high-quality training is further shown by the fact that participants with prior CPR training reported being better able to perform a variety of CPR techniques than those without training.

Laypeople must be able to do efficient, rapid CPR after successfully identifying a person in CA. Despite the fact that over half (59.5%) of the participants claimed to be capable of doing chest compressions appropriately, less than a quarter of the respondents reported being able to perform CPR effectively. Practical CPR skills understanding was really low in this study. Nonetheless, those who had received CPR training were noticeably more informed than those who had not, a result consistent with previous research.^21,27^ Ability to open the airway, perform mouth-to-mouth ventilation and perform chest compressions were significantly associated with BLS training. This finding is supported by other studies which showed that CPR training increases skills acquisition and retention in performing chest compressions. In comparison to other aspects of CPR, proper airway management and breathing abilities, as well as chest compression fraction, are considerably more dependent on training level.^31^ A study in Germany found that before training, 29.5% of students could perform chest compressions compared to 99.2% post-training.^32^

Creating a care system that could reduce mortality and enhance OHCA outcomes requires knowledge of the attitudes and barriers to performing rapid CPR, given the crucial role bystander CPR plays in OHCA survival. Compared to another study, a significant proportion of respondents (four in every ten) reported having seen abrupt CA. There is a strong need to enhance laypeople’s CPR knowledge and abilities through high-quality training so they can respond to OHCA in light of this finding and another Johannesburg study that also found a high rate of observed OHCA.^18^ Most participants would prefer to provide CPR to friends and relatives rather than to strangers, which is consistent with other research.^20,21,24,33^ Bystanders may be reluctant to provide CPR for a number of reasons. According to the current findings, prior research has shown that the most significant issues with bystanders initiating CPR are insufficient knowledge and skills and fear of making mistakes.^20,21,34^ However, another study of South African student-teachers revealed that the biggest obstacle was their fear of being held legally responsible.^24^ It has been demonstrated that CPR training increases laypeople’s proficiency and self-assurance when performing CPR, hence lowering their anxiety of making a mistake.^13,17^ Notably, a considerable proportion of participants (74.7%) expressed willingness to undergo CPR training. This is consistent with the results of another study carried out in South Africa.^24^ This can serve as a solid starting point for initiatives meant to raise laypeople’s CPR knowledge and proficiency in South Africa.

Almost three-quarters of the respondents had never heard of an AED, and a minimal number had seen it and also knew what it is used for. Nevertheless, the results of this investigation do not support a previous study which found that 97% of participants correctly identified the defibrillator as a device that is able to save the patient’s life using an electric shock.^35^ This high percentage may be attributed to AED availability in over 830 public places across the city of Vienna in Austria, most of which are open 24 h a day, 7 days a week.^35^ The reasons for poor AED awareness could not be determined from the present study and warrant further investigation. Additionally, respondents in this study were drawn from a single faith-based setting, and the medical, paramedical and health sciences students were excluded, whereas in the Vienna study, respondents were randomly selected from the Viennese population with no specific exclusion criteria.

### Implications of the study

The study shows that lay churchgoers have substantial gaps in their BLS knowledge, attitudes and practices, with untrained lay believers having poorer competence. These results suggest that, given high church attendance, BLS education should focus on faith-based communities. Church programmes that incorporate BLS training could improve community readiness for cardiac emergencies, decreasing bystander hesitancy and increasing survival rates.

### Strengths and limitations

Limitations of this study include the use of a cross-sectional design, a single-site setting, convenience sampling, self-reporting data collection and response bias. Given that the study was only done in one location, the results of the study can only be used with caution when generalising. Also, while the questionnaires used in this research were based on two previously validated instruments, the reliability of the test was never established. The number of participants in the study was lower than the intended number of 270, with only 220 people consenting to participate. This may have reduced statistical power to detect some association, and the findings should be interpreted with this consideration. One of the strengths of the study is that there was involvement of a heterogeneous church group consisting of individuals from different backgrounds within Johannesburg. The study offers valuable data at the community level regarding CPR training and BLS practice by laypersons.

## Conclusion

Given that one-fourth of the respondents had witnessed a CA and considering the significance of CPR in OHCA, the study participants’ limited knowledge is concerning. However, compared to individuals without prior CPR training, participants who had received such training reported being better at performing different CPR techniques. The results indicate that there is a need for increasing CPR training and education among laypersons in church communities, as previous CPR training was associated with better self-reported BLS practice. It is encouraging that participants were willing to be trained, which provides an opportunity to explore the most effective and efficient modes of training for layperson CPR in South Africa. Further research is needed to identify the best strategy to ensure the lay populace is equipped to help increase the survival rates of OHCA and ensure more AEDs are placed in public places.
